# Induction of proline-rich proteins in response to tannin treatment in *Caenorhabditis elegans*

**DOI:** 10.1038/s41598-025-11651-1

**Published:** 2025-08-11

**Authors:** Luise Greiffer, Luka Ressmann, Charlotte Sophia Kaiser, Eva Liebau, Verena Spiegler

**Affiliations:** 1 Institute for Pharmaceutical Biology and Phytochemistry, University of Münster, Münster, Germany; 2 Institute of Integrative Cell Biology and Physiology, University of Münster, Münster, Germany

**Keywords:** Ecology, Molecular biology

## Abstract

Salivary proline-rich proteins (PRPs) represent a common mechanism of defense against tannins in mammals. Few reports exist regarding the occurrence or PRPs with similar function in nematodes and none of these proteins or their coding genes have been functionally characterized so far. In *Caenorhabditis elegans*, two genes (*clx-1* and *T22D1.2*) were strongly induced upon tannin treatment of the nematodes, both of them potentially encoding proline-rich proteins. Therefore, translation of these genes into proteins was confirmed and the expression pattern was investigated in more detail. Particularly *T22D1.2* was found to be exclusively up-regulated in worms treated with test substances possessing astringent properties, especially tannins, whereas no expression was observed for any other stressor or in the untreated control group. Similar to mammalian PRPs, repetitive proline-rich sequences were identified in both of the corresponding proteins. A potential role in tannin defense was supported by an increased survival of tannin-treated worms when *T22D1.2* was constitutively expressed under the *vit-5* promoter. However, no differences were observed in the *clx-1* and *T22D1.2* knockout mutants in comparison to the wild type, respectively. Within the current study, evidence was provided for the existence of repetitive proline-rich proteins in the free-living nematode *C. elegans*, of which particularly *T22D1.2* may be involved in tannin defense.

**Supplementary Information:**

The online version contains supplementary material available at 10.1038/s41598-025-11651-1.

## Introduction

Tannins comprise a group of secondary metabolites that is widespread across the plant kingdom. Structurally, proanthocyanidins (syn. condensed tannins) and hydrolyzable tannins (HT) form two major subclasses found in terrestrial plants^1,2^: Proanthocyanidins (PAC) consist of flavan-3-ol units that are mainly linked via C-4 to C-6 or C-8 of another monomeric unit^1^. Hydrolyzable tannins, on the other hand, are formed by gallic acid and its derivatives esterified with glucose or other polyols^2^. Despite their structural differences, both, PAC and HT share the property of binding to proteins leading to similar biological effects. While this activity is desired in medicinal applications of extracts from tannin-rich plants^3^, the intake of tannins by mammalian herbivores can affect nutrient availability due to interfering with digestive enzymes^4^. At higher doses or in susceptible animal species, it is associated with adverse effects, such as damage to the intestinal mucosa and epithelium as well as kidney or liver failure^5,6^.

Such detrimental activities can be circumvented either by avoiding tannin-rich plant species as feed or by counteracting their effects. Salivary proteins, and particularly proline-rich proteins (PRP), that bind tannins with high affinity represent one such countermeasure^6–8^. In line with a role as a mechanism of defense, mammals like browsers, feeding on a tannin-rich diet, generally tend to produce higher amounts of proline-rich proteins in the salivary glands compared to species consuming less tannins, e.g., grazers^6,9,10^. Similarly, PRPs are completely absent in *Theropithecus gelada*, a graminivorous baboon species, while omnivorous hamadryas baboons possess PRPs^11^. However, a high content of proline itself is not necessarily indicative of the affinity of a protein to bind tannins^10^. And likewise, herbivorous species lacking PRPs are found to secrete structurally distinct proteins with high affinity for tannins^12^. Compared to mammals, the effects of tannins are frequently much less pronounced in non-mammalian herbivores. In several insect species, neither toxic nor feeding deterrent effects were observed, whereas in some, pupal growth and survival of larvae and caterpillars were negatively affected by tannin consumption^13,14^. Similarly, condensed tannins had a negative impact on the occurrence and density of certain herbivore insects, but not for the majority of species^15^.

Regardless of their exposure and adaption to tannins, insects possess various protective mechanisms, foremost surfactants that prevent protein-precipitating activities of tannins in the midgut^13,16^. The secretion of the amino acid glycine into the digestive juice represents another mode of defense against protein denaturing plant secondary metabolites^17^. Further properties include the maintenance of a high midgut pH to inhibit tannin-protein binding as well as different antioxidative mechanisms. Additionally, the peritrophic envelope acts as a barrier protecting the midgut epithelium and preventing the absorption of tannins^13^.

Finally, plant pathogenic nematodes represent the third group of herbivores. Surprisingly, only few studies report on the role of tannins in nematode resistance, despite the substantial impact of plant parasitic nematodes in agriculture on the one hand, and the plethora of secondary metabolites produced in response to nematode infestation on the other^18^. One example is a banana cultivar with high levels of condensed tannins and a distinct tannin composition compared to other cultivars, which conferred resistance against the nematode *Radopholus similis*^18,19^. Phenolic compounds, including tannins, have also been associated with plant resistance towards the root lesion nematodes of the genus *Pratylenchus*^20^. Interestingly, a study focusing on the identification of effector genes in *Pratylenchus penetrans* revealed the presence of transcripts encoding proteins extremely rich in proline^21,22^. Also, glycine was highly abundant in the protein sequences predicted from the gene transcripts localized in the esophageal glands^22^. Particularly the predicted proline-rich proteins were hypothesized to act as a countermeasure against tannin-like deposits produced by the plant during *P. penetrans* infection^21^. Two genes (*T22D1.2* and *clx-1*) also predicted to code for repetitive proline-rich proteins were found to be strongly induced in the free-living nematode *Caenorhabditis elegans* upon treatment with a plant extract and a fraction enriched in condensed tannins^23^. Although still little is known about the ecology of *C. elegans*, this species is surrounded by decomposing organic materials such as fruits or stems in its natural habitat, providing bacteria as food source^24–26^. Fruits, in turn, are common sources of tannins^27,28^ and the nematode may be exposed to tannins derived from rotting plant material^29^ in its surroundings. It is therefore conceivable that the two hypothetical proline-rich proteins represent a protective mechanism against tannins, however, their function remains unclear. Aim of the current study was therefore, to confirm the presence of PRPs in *C. elegans* and to investigate their potential role in tannin defense.

## Materials and methods

### Chemicals and reagents

If not stated otherwise, chemicals were purchased from AppliChem (Darmstadt, Germany). A hydroethanolic extract from the leaves of *Combretum mucronatum* Schumach. & Thonn. (CM) and its PACs, procyanidin B2 (purity 96%, HPLC-UV) and procyanidin C1 (purity 95%, HPLC-UV), were obtained as described previously^30^. The acetone-water (7:3) extract from the aerial parts of *Phyllanthus urinaria* L. (PU) together with its major ellagitannin, geraniin (purity 98%, HPLC-UV), were prepared as described by Jato et al.^31^.

### *C. elegans* strains and culture conditions

Wild-type (WT) *C. elegans* (N2 Bristol strain), RG3081 *clx-1*(ve581) IV^32^ and GE24 *pha-1* (e2123) III were obtained from the Caenorhabditis Genetics Center (University of Minnesota) and were maintained on nematode growth medium (NGM) agar with *Escherichia coli* (strain OP50) as food source^33,34^. *C. elegans* strains created in our laboratories are listed in Table 1.

**Table 1:** Overview of the *C. elegans* strains generated for this study.

Function	Strain	Genotype
GFP fusion	WWU202	*evaEx104[clx-1p::clx‐1::gfp* + *pha‐1(+)]*
	WWU203	*evaEx106[T22D1.2p::T22D1.2::gfp* + *pha-1(+)]*
	WWU204	*evaEx106[T22D1.2p::gfp* + *pha-1(+)]*
	WWU205	*evaEx106[vit-5p::T22D1.2::gfp* + *pha‐1(+)]*
	WWU701	*evaEx101* [*T22D1.2p::gfp* + *rol*−6(su1006)]
Knockout	WWU207	*clx-1(ve581) IV; T22D1.2(eva102[Δ55‐1185]) IV*
	WWU702	*T22D1.2(eva102[Δ37-1185]) IV*

All strains were kept at 20 °C, except for *pha-1* mutants that were grown at 15 °C or at 25 °C after *pha-1* rescue^35,36^.

Age synchronous cultures were obtained by hypochlorite treatment of adult hermaphrodites. Briefly, worms were rinsed off the agar plates in M9 buffer and after removal of the supernatant, the worm pellet was treated with alkaline hypochlorite solution (1.7 mL water, 0.2 mL sodium hypochlorite (Carl Roth, Karlsruhe, Germany), 0.1 mL 10 M NaOH) for 7 min with constant agitation. The solution was then centrifuged at 4500 × *g* for 1 min and after removal of the supernatant, the egg pellet was washed at least three times with M9 buffer^37^.

### Generation of vectors

For the construction of reporter gene plasmids, gene sequences were amplified by PCR from genomic DNA (Gene Jet Genome DNA Purification Kit, Thermo Scientific, Waltham, USA) using Phusion^®^ High-Fidelity DNA polymerase (Thermo Scientific, Waltham, USA). PCR products were cloned into the respective vectors by ligation (T4 DNA ligase (Thermo Fisher Scientific)) or using the In-Fusion HD Cloning Kit (Clontech, Takara Bio, USA) according to standard protocols. All primer sequences are listed in Supplementary Table S1.

#### clx-1p::clx‐1::gfp and clx-1p::gfp constructs

A promoter fragment including 2000 bp of the upstream region and the complete coding sequence of *clx-1* was cloned into pPD95.77 kindly provided by A. Fire (Carnegie Institute, Baltimore, MD). Additionally, promoter fragments including approx. 2000 bp, 1500 bp, 1000 bp, 350 bp–250 bp upstream of *clx-1* without the sequence of *clx-1* were fused to GFP.

#### T22D1.2p::T22D1.2::gfp and various T22D1.2p::gfp constructs

A promoter fragment containing 2000 bp of the upstream region and the complete coding sequence of T22D1.2 was amplified and cloned into pPD95.77. The start codon for GFP was subsequently removed from the plasmid by site directed mutagenesis as described below.

Strain WWU701 *evaEx101* [*T22D1.2p::gfp* + *rol*−6(su1006)] was generated as described previously^23^. In addition, promoter fragments of approx. 1500 bp, 350 bp and 250 bp upstream of T22D1.2, respectively were cloned into pPD95.77.

#### vit-5p::T22D1.2::gfp

The coding sequence of T22D1.2 was amplified and cloned into pPD49.83 carrying a promoter fragment of 2000 bp upstream of *vit-5*^38^. The start codon of GFP was subsequently removed from the plasmid by site directed mutagenesis.

Site directed mutagenesis was performed using QuickChange II XL Site-Directed Mutagenesis Kit (Agilent Technologies, Santa Clara, USA) to remove the start codon of the *gfp* coding sequence from pPD95.77 or pPD49.86 as described above, as well as to insert sgRNA targeting *T22D1.2* into vector pDD162. To generate the *clx-1*,*T22D1.2* double knockout mutant, sgRNA targeting *T22D1.2* was introduced into plasmid pRB1017 as described by Arribere et al.^39^. Guide sequences were designed using the online tool CRISPOR^40^.

### Generation of transgenic *C. elegans*

Transgenic *C. elegans* lines were established by microinjection using pBX (temperature selection) as selection marker. Reporter gene plasmids were microinjected into the germline of young adult *pha-1* mutants together with selection marker plasmid pBX^36^. Germline transformation of GE24 *pha-1* (e2123) for the generation of *T22D1.2* knockout mutants was performed by microinjection of plasmid pDD162 (50 ng/µL) containing the sgRNA targeting *T22D1.2*, together with pJW1285 (kind gift from Jordan Ward (Addgene plasmid # 61252))^35^ and a repair template for *pha-1* co-conversion^35^. Germline transformation of RG3081 *clx-1(ve581)* was performed by co-injection of pDD162, pJA58 (kind gift from Andrew Fire (Addgene plasmid # 59933))^38^, pRB1017 containing *T22D1.2* targeting sgRNA and repair templates to edit the *dpy-10* (CACTTGAACTTCAATACGGCAAGATGAGAATGACTGGAAACCGTACCGCATGCGGTGCCTATGGTAGCGGACTTCA CATGGCTTCAGACCAACAGCCTAT^39^) and *T22D1.2* locus, respectively. Inclusion of a restriction site for *Bam*HI facilitated genotyping^35^ of the roller F1 progeny and of the F2 progeny showing a wild-type phenotype, respectively^39^. Genotyping PCRs were performed using single-worm lysate as DNA template. Briefly, 3 µL lysis buffer (10 mmol/L TRIS pH 8.0, 50 mmol/L KCl, 2.5 mmol/L MgCl_2_, 0,45% Tween^®^ 20, 0.05% gelatin, 1 mg/mL proteinase K) containing a single worm were snap frozen in liquid nitrogen and lysed at 65 °C for 1 h, followed by proteinase K inactivation at 95 °C for 15 min. PCR amplification was carried out using DreamTaq™ DNA Polymerase (Thermo Scientific, USA) with 0.5 µL of DNA template. 5 µL of PCR product were used for *Bam*HI digestion (FastDigest restriction enzymes, Thermo Scientific, USA). DNA sequences were additionally determined for PCR products from homozygous F2 progeny (Eurofins Genomics, Ebersberg, Germany). Primer sequences are listed in supplementary Table S1.

### Isolation and identification of GFP fusion proteins

CLX-1 and T22D1.2 fused to GFP were isolated from young adult worms of strains WWU202, WWU203 and WWU205. Unlike WWU205, it was necessary to induce protein expression in WWU202 and WWU203 by incubating the animals for 18 h in a solution of CM (200 µg/mL), followed by three washing steps with M9 buffer to remove residual extract solution. Worms were pelleted, frozen in liquid nitrogen and lysed in RIPA buffer (10 mmol/L TRIS buffer pH 7.5, 150 mmol/L NaCl, 0.5 mmol/L EDTA, 1% Triton™ X-100, 0.1% sodium dodecyl sulfate (SDS), 1% sodium deoxycholate) including 1 µL protease inhibitor cocktail (P8849, Sigma-Adrich, Germany) using a Minilys^®^ homogenizer (Bertin Instruments, Montigny-le-Bretonneux, France). 300 µL of dilution buffer (10 mM Tris/Cl pH 7.5, 150 mM NaCl, 0.5 mM EDTA) for subsequent use with GFP-Trap^®^ Magnetic Particles M-270 (ChromoTek, Planegg-Martinsried, Germany) were added to the lysate, followed by centrifugation at 4 °C for 20 min at 16,000 × *g* to remove debris. The protein concentration was determined as described by Bradford^41^ with bovine serum albumin as standard. The GFP fusion proteins were purified from the lysates using GFP-Trap^®^ Magnetic Particles M-270 according to the manufacturer’s protocol using the SDS-sample buffer for elution. The respective fusion proteins were detected by SDS-PAGE, followed by Western blot analysis using a rabbit anti-GFP primary antibody (1:5000; Dianova, Hamburg, Germany) and alkaline phosphatase-conjugated donkey anti-rabbit secondary antibody (1:10000; Dianova).

Purified proteins were identified by mass spectrometry (MS) at the *Mass spectrometry-based Proteomics Unit Biology of Plants* (MSPUB) of the University of Münster according to standard procedures described previously^38^. Samples were prepared from gel slices after destaining and protease digestion according to established protocols^42^ as previously described^38^. For each sample, the most appropriate protease was selected, i.e., elastase was used for CLX-1::GFP, T22D1.2::GFP (from WWU203) was trypsinized, and T22D1.2::GFP (from WWU205) was first digested by LysC and then by pepsin. For protein identification, MS data were matched to the target sequences of the respective fusion proteins using MS Fragger^43^, SearchGUI^44^ and MaxQuant^45^. Peptide sequences obtained for each strain are given as supplementary material.

### Treatment of GFP reporter strains with different stressors

Young adult worms (strains WWU701, WWU202 and WWU204) expressing *T22D1.2p::gfp* or *clx-1p::clx‐1::gfp* were treated for 6–18 h with different stressors, respectively. Sodium chloride (300 mM), cadmium chloride (8 mM) and juglone (250 µM; Sigma Aldrich, Taufkirchen, Germany) were added to NGM agar to achieve the respective concentrations. Heat stress (30 °C) was applied to worms kept on regular NGM plates. Tunicamycin (5 µg/mL; Sigma Aldrich, Taufkirchen, Germany), Epicatechin (1 mM; Carl Roth, Karlsruhe, Germany), Geraniin (200 µg/mL), Procyanidin C1 (1 mM), Procyanidin B2 (1 mM), as well as the plant extracts PU (200 µg/mL) and CM (2–2000 µg/mL) were solubilized in DMSO and diluted in M9 buffer to the given concentrations. Salts of divalent cations were tested in an 80 mM solution of NaCl at the highest concentration that was tolerated by the worms: 37.5 µM Al_2_(SO_4_)_3_, 1.25 mM NiSO_4_, 2.75 mM ZnCl_2_, 3.34 mM CdCl_2_ and 0.25 mM CuSO_4_. Worms incubated on regular NGM plates served as negative control. For the assay in liquid medium, a negative control containing 1% DMSO, corresponding to the highest concentration, was included. In addition to young adults, larval stages L1 to L4 of strain WWU203 were treated with CM (200 µg/mL) for 24 h. GFP expression in L4 larvae (WWU203) was additionally monitored after 3 and 6 h. Experiments were performed in triplicate with at least 10 animals for each treatment per biological replicate.

### Knockdown of selected transcription factors

RNAi mediated knockdown was performed in *T22D1.2p::gfp* worms following established protocols^46^. Age synchronous L1 larvae were grown at 25 °C on NGM agar plates containing 50 µg/mL ampicillin and 2.5 mM iso-propyl-β-d-thiogalactopyranoside (IPTG) that were supplemented with *E. coli* HT115, until the nematodes reached young adult stage. The *E. coli* strain HT115 produced double-stranded RNA of *daf-16*, *skn‐1* and *pha‐4*, respectively. After incubation on RNAi plates, worms were transferred to M9 buffer containing 0.2 mg/mL CM and treated for 3 h to induce expression of GFP. Experiments were performed in triplicate with at least 10 animals for each treatment per biological replicate.

### Confocal laser scanning microscopy

Fluorescence in GFP reporter strains was determined in the dark by confocal laser scanning microscopy (LSM 510, Carl Zeiss, Jena, Germany). An argon laser was use for excitation at 488 nm. The emission (HFT 488) was split by secondary dichroic beamsplitter NFT 545 and was detected using a 505–550 nm bandpass filter. For strains WWU202 and WWU204 treated with CM at 0.2 and 2 mg/mL, the pinhole was set to 1 Airy unit and the detector gain was reduced from 500 to 350. These settings were also applied to assess treated and untreated strains carrying *clx-1* and T22D1.2 promoter fragments of varying length. For the *clx-1* promoter experiment, images obtained under both acquisition conditions, i.e., setting the gain to 500 or to 350 with reduction of out-of-focus light, respectively, were included as Supplementary Data for comparison. For further analysis, in addition to detection of 505–550 nm (green), the emission above 650 nm (autofluorescence, red) was recorded and subtracted from the GFP signal during image acquisition. Images were taken at a resolution of 2048 × 2048 px and a scan speed of 6. Fluorescence intensities were quantified using ImageJ 1.50i^47^ and reported in relation to the respective control group. Prior to microscopy, nematodes were transferred to an agarose pad (1% agarose) on a microscope slide and immobilized in levamisole hydrochloride solution (100 mmol/L).

### Quantitative real-time PCR (RT-qPCR)

Levels of *clx-1* and *T22D1.2* transcripts were quantified by RT-qPCR after treatment of L4 larvae with 0.2 mg/mL CM in M9 buffer for 30 min, 2 h and 24 h, respectively. An additional group of worms was treated for 2 h, washed with M9 buffer to remove residues of the plant extract, and was then allowed to recover on NGM plates for another 24 h. For every treatment, a corresponding negative control receiving 0.1% DMSO, equivalent to the DMSO content in the CM solution, was included. Prior to RNA isolation, all samples were thoroughly washed at least three times with M9 buffer. All following steps were performed as described previously^23^. The NucleoSpin^®^ RNA, Mini Kit (Macherey-Nagel, Düren, Germany) was used for RNA isolation, Y45F10D.4 and *pmp-3* were used as housekeeping genes^48^. All primer sequences are listed in Table S1. Experiments were performed in at least three independent replicates.

### *C. elegans* mortality after tannin treatment

The susceptibility towards tannin treatment was assessed in *C. elegans* strains WWU202, WWU203, WWU205 and WWU207 compared to the wild-type as control. Age synchronous young adult worms were rinsed off NGM agar plates and were washed in M9 buffer. Ten to twenty animals were transferred to a 48-well plate containing CM solutions in a concentration range of 0.02 to 5 mg/mL and 5 µL of *E. coli* OP50 as food source in a volume of 250 µL per well. To avoid bacterial growth during the assay and interaction with the test samples, overnight cultures were repeatedly frozen in liquid nitrogen, followed by incubation at 70 °C for 10 min. The bacterial suspension was centrifuged at 6500 × *g* for 1 min, the supernatant was discarded and the pellet was resuspended in M9 buffer. A solution of 2% DMSO, corresponding to the highest DMSO concentration used in the assay, served as negative control. After 48 h of incubation at 25 °C, survival was assessed by counting the number of dead versus living worms in each well. Immotile nematodes were considered dead if their bodies were fully straight and they did not respond to touch with an eyelash. Strains that were usually maintained at 20 °C were cultured at 25 °C for at least 14 days prior to the test to avoid additional stress due to the elevated temperature. All assays were performed in three independent experiments with four technical replicates per treatment.

### Statistical analysis

Statistical evaluation was performed using GraphPad Prism^®^ 10 (GraphPad Software, Inc., La Jolla, CA, USA). Differences among mean values were determined by ANOVA with Bonferroni post-test. *P* values < 0.05 were considered significant.

## Results

**Fig. 1:**
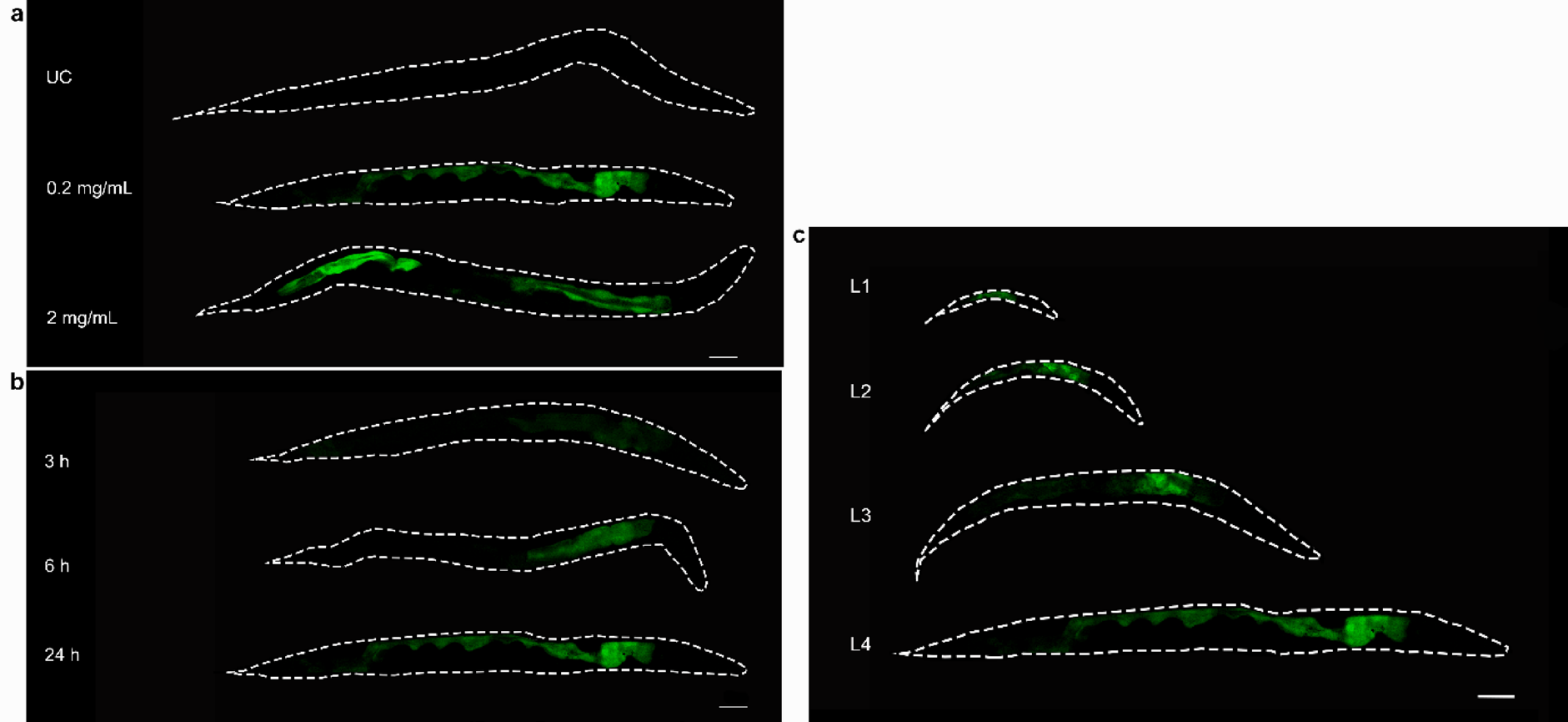
Representative images of *C. elegans* strain WWU203 after incubation with CM in M9 buffer. (**a**) Concentration dependent expression after 24 h and (**b**) time dependent expression of T22D1.2::GFP at 0.2 mg/mL CM. (**c**) Expression of T22D1.2::GFP was observed in all larval stages after treatment with CM at 0.2 mg/mL for 24 h. UC: Untreated control. Scale bar 50 μm. Experiments were performed in triplicate with at least ten animals per replicate.

Two genes, *clx-1* and *T22D1.2* were previously found to be strongly up-regulated in *C. elegans* in response to condensed tannins and are hypothesized to play a potential role in tannin defense^23^. Therefore, the first step in characterizing their function was to evaluate their expression pattern in more detail. Due to its unique sequence and strong up-regulation in previous experiments^23^, our primary focus was on *T22D1.2*. In the first step, a time and concentration-dependent up-regulation of the gene was observed in *C. elegans* L4 larvae expressing a GFP fusion protein of *T22D1.2*. The animals were stressed for up to 24 h with a plant extract from *Combretum mucronatum* (CM), which had previously been characterized and is known to contain large amounts of condensed tannins^30^. As shown in Fig. 1a, no fluorescence was observed in the untreated control group, whereas tannin treatment at different concentrations led to expression of the fusion protein in the intestine. In animals receiving the same treatment, the fluorescence intensity increased with the time of exposure to CM (Fig. 1b). In addition, the expression of *T22D1.2* in different developmental stages was investigated and fluorescence was detected in the intestine of all treated larvae (Fig. 1c).

**Fig. 2:**
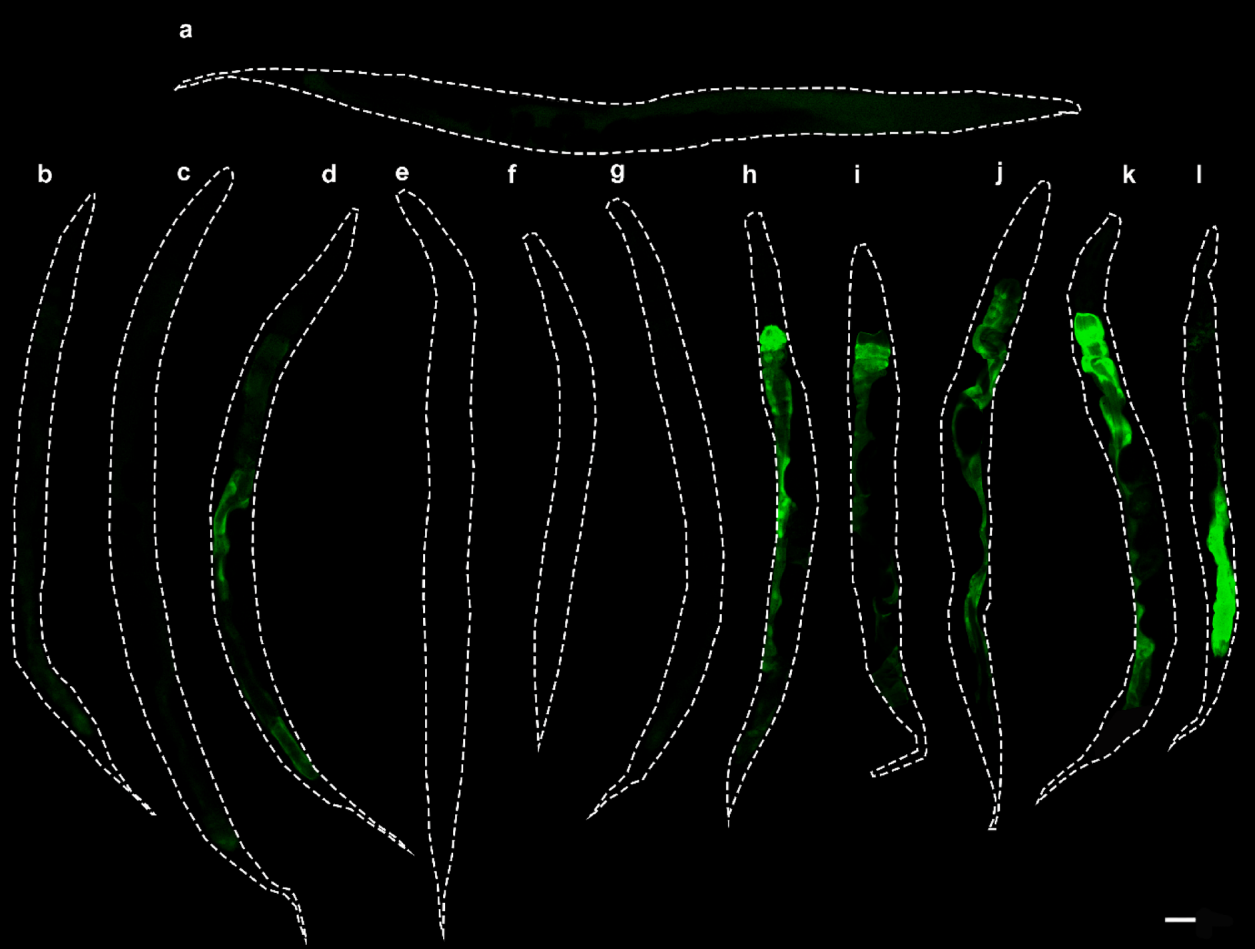
*C. elegans* strain WWU204 expressing GFP under control of the *T22D1.2* promoter after treatment with different stressors for 18 h. Representative GFP fluorescence images of the respective animals: (**a**) Untreated control, (**b**) heat stress (30 °C), (**c**) osmotic stress (300 mM NaCl), (**d**) heavy metal stress (8 mM CdCl_2_), (**e**) oxidative stress (250 µM juglone), (**f**) untreated control in M9 buffer including 1% DMSO, (**g**) endoplasmic reticulum stress (5 µg/mL tunicamycin), (**h-j**) *C. mucronatum* extract at 0.02 mg/mL, 0.2 mg/mL and 2 mg/mL, (**k**) Geraniin (0.2 mM), (**l**) *P. urinaria* extract (0.2 mg/mL). Scale bar 50 μm. Experiments were performed in triplicate with at least ten animals per replicate.

In the next step, young adult worms were incubated with a selection of common stressors in addition to CM to assess whether the expression of *T22D1.2* could be part of a general stress response or is induced more specifically by the treatment with tannins. Strain WWU204 carrying a promoter-GFP fusion showed stronger fluorescence and was therefore used in this experiment instead of WWU203. As shown in Fig. 2, T22D1.2 was not expressed at all in the untreated control (Fig. 2a), but was markedly induced by the treatment with CM (Fig. 2h-j). Regarding the expression in worms incubated on NGM agar plates with non-tannin stressors, weak fluorescence could only be detected in animals treated with the heavy metal cadmium chloride (8 mM; Fig. 2b-g).

**Fig. 3:**
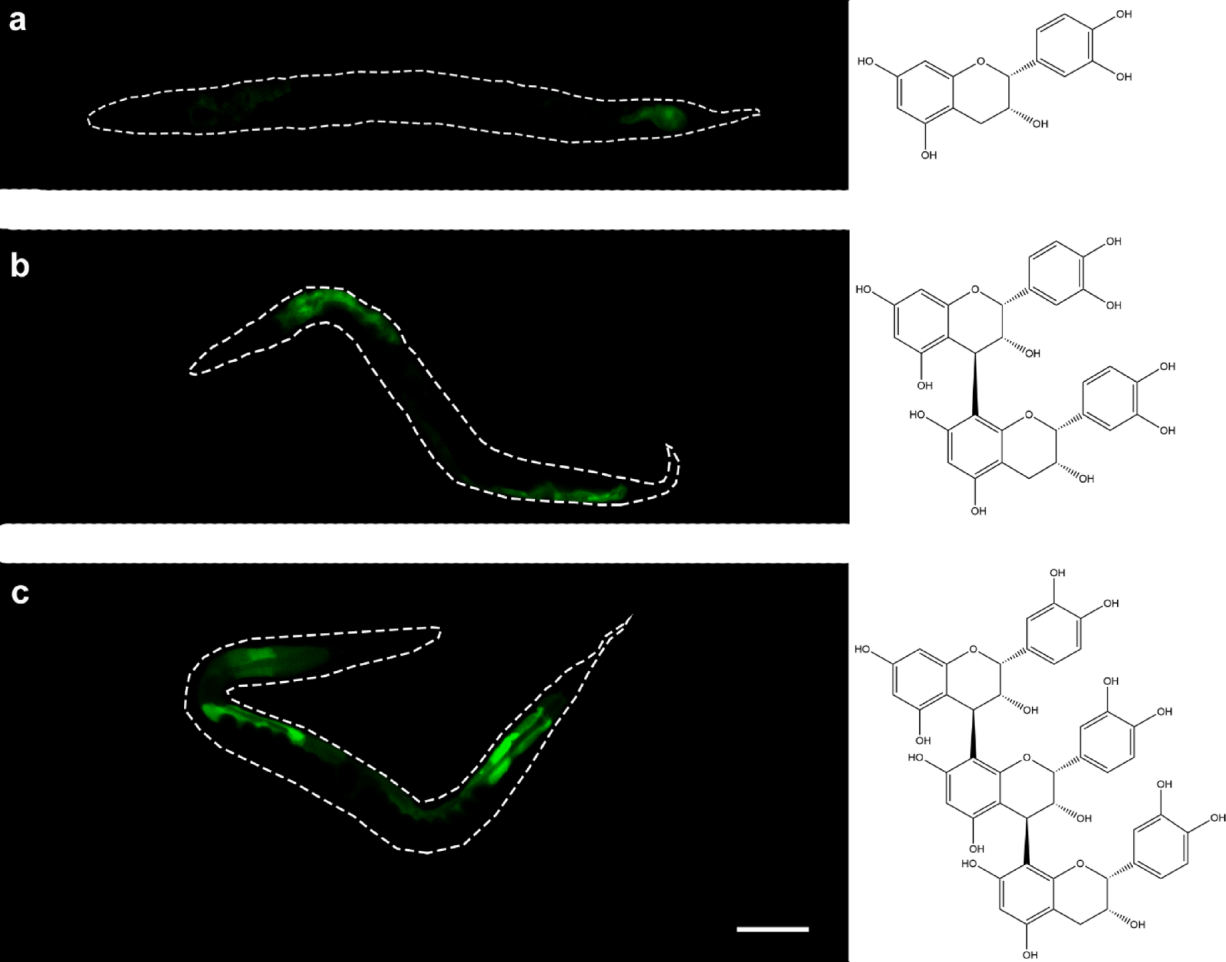
Representative images of *C. elegans* strain WWU701 expressing *pT22D1.2::gfp* after treatment with (**a**) epicatechin, (**b**) procyanidin B2 and (**c**) procyanidin C1 for 6 h in M9 buffer. Structures depicting the respective compounds are given on the right. Scale bar 100 μm. Experiments were performed in triplicate with at least ten animals per replicate.

To determine whether astringent but structurally unrelated compounds cause the same induction of GFP expression, a phytochemically characterized extract of *Phyllanthus urinaria* (PU) together with its major constituent, the ellagitannin geraniin, was studied in the same way. In line with the hypothesis that T22D1.2 expression is induced by astringent compounds, PU as well as geraniin also resulted in significantly fluorescent worms (Fig. 2k, l). To further corroborate the assumption that the expression of T22D1.2 is linked to the astringent properties of the tannins, structurally related flavan-3-ol derivatives of increasing size were tested in the same assay. Fluorescence was again barely detectable in worms treated with epicatechin (Fig. 3a). Epicatechin represents the monomeric unit of PAC found in CM and was expected to have the lowest ability to precipitate proline-rich proteins among the three compounds^49^. In line with previous findings concerning the interaction with salivary proteins^49^, GFP expression increased with molecular size, i.e., with an increasing number of flavan-3-ol units and thus with increasing astringency (Fig. 3b and c).

**Fig. 4:**
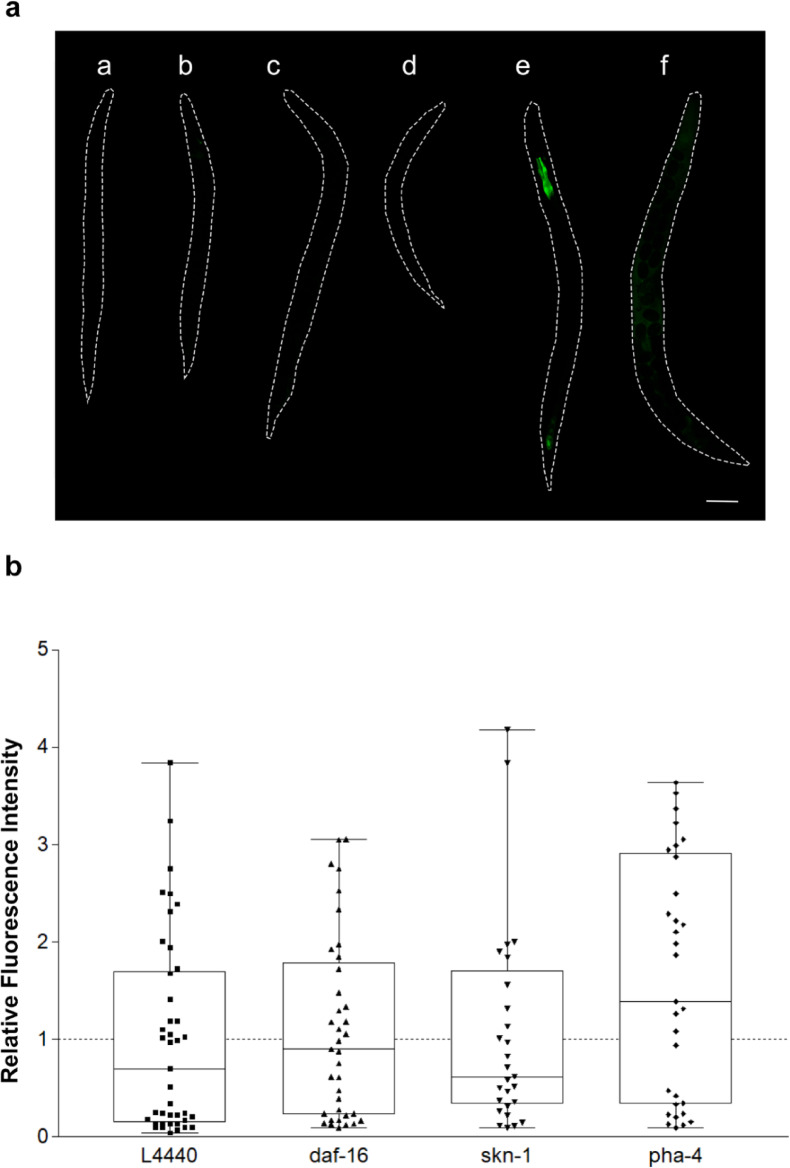
Expression of *pT22D1.2::gfp* in *C. elegans* strain WWU204. (**a**) Representative images of worms after 18 h treatment with salts of different multivalent cations (b: 37.5 µM Al_2_(SO_4_)_3_; c: 1.25 mM NiSO_4_; d: 2.75 mM ZnCl_2_; e: 3.34 mM CdCl_2_; f: 0.25 mM CuSO_4_). a: untreated control. Tested concentrations correspond to the highest concentrations tolerated by the worms. Scale bar 200 µM. (**b**) GFP expression during RNAi mediated knockdown of transcription factors *daf-16*, *skn-1* and *pha-4* in worms treated with 0.2 mg/mL CM. *E. coli* HT115 containing the empty vector L4440 served as control with its fluorescence intensity set to 1. Boxes represent median values with upper and lower quartile, whereas each data point represents the fluorescence of one animal. Experiments were performed in triplicate with at least ten animals per replicate.

The observation that treatment with CdCl_2_ also induced weak fluorescence, prompted us to investigate the effect of other multivalent metals in strain WWU204, as metal ions are also generally capable to cause an astringent perception^50^. Besides cadmium chloride, GFP expression was only detectable in worms treated with copper sulfate (Fig. 4a). This was unexpected, because aluminum and zinc salts have also been reported to cause astringency in humans^51–53^.

To further confirm that the induction of *T22D1.2* is not part of a general stress response, GFP expression was monitored in strain WWU204 during RNAi-mediated knockdown of selected transcription factors involved in various stress-related pathways. Neither knockdown of *daf-16*, *skn-1* nor *pha-4* resulted in altered expression of the fusion protein in CM treated nematodes (Fig. 4b), corroborating the idea that the up-regulation of this proline-rich protein is linked to the astringent properties of the tannins and not controlled by typical stress related transcription factors.

**Fig. 5:**
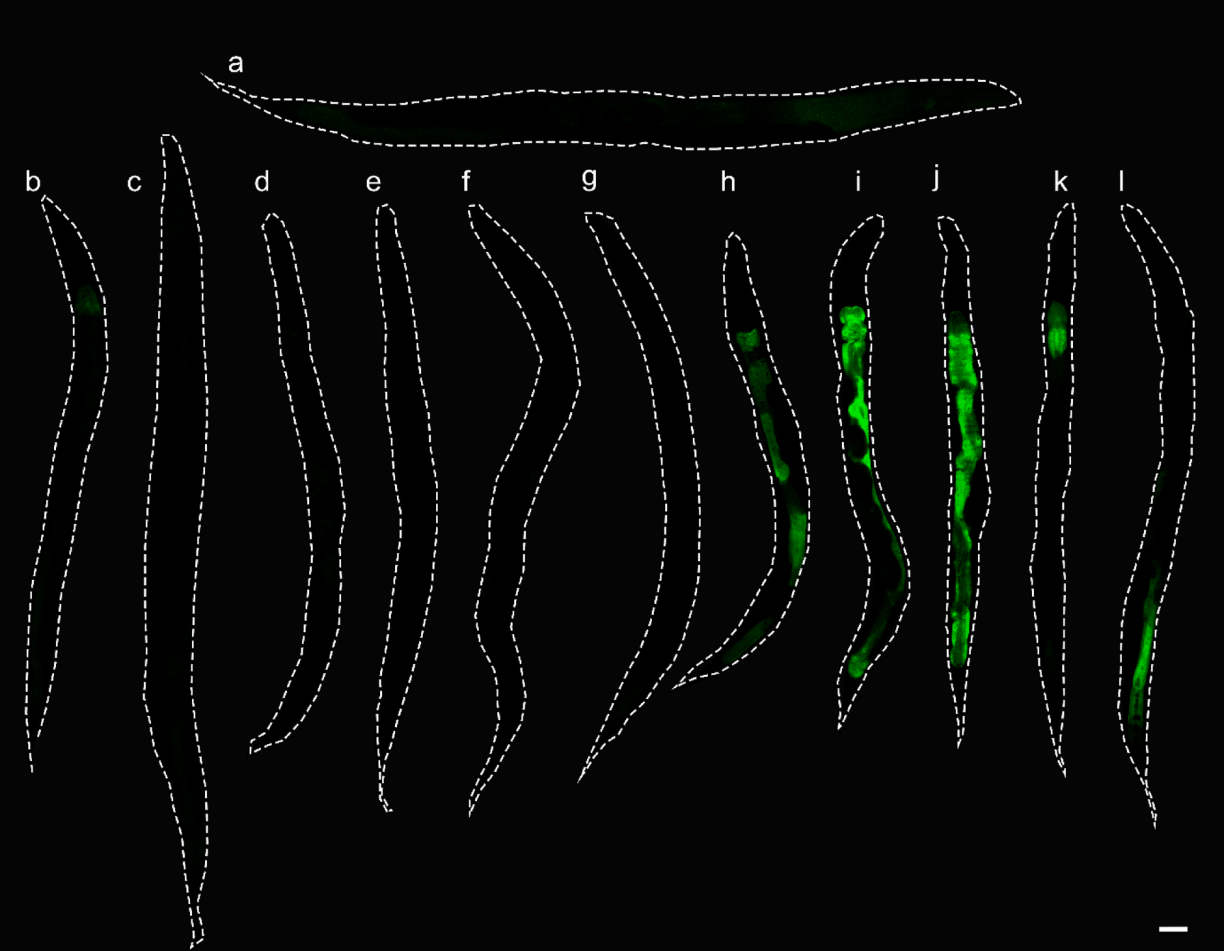
*C. elegans* strain WWU202 expressing CLX-1::GFP after treatment with different stressors for 18 h. Representative GFP fluorescence images of the respective animals: (**a**) UC: Untreated control, (**b**) heat stress (30 °C), (**c**) osmotic stress (300 mM NaCl), (**d**) heavy metal stress (8 mM CdCl_2_), (**e**) oxidative stress (250 µM juglone), (**f**) untreated control in M9 buffer including 1% DMSO, (**g**) endoplasmic reticulum stress (5 µg/mL tunicamycin), (**h-j**) *C. mucronatum* extract at 0.02 mg/mL, 0.2 mg/mL and 2 mg/mL, (**k**) Geraniin (0.2 mM), (**l**) *P. urinaria* extract (0.2 mg/mL). Scale bar 50 μm. Experiments were performed in triplicate with at least ten animals per replicate.

Similar to *T22D1.2*, the expression of a CLX-1::GFP fusion protein was investigated in the presence of different stressors. Again, an induction of protein expression was detected exclusively in response to the treatment with tannins (Fig. 5). The results obtained from the reporter fusion experiments were further corroborated by RT-PCR, where the number of transcripts in the untreated control was either very low (*clx-1*) or even below the threshold of detection (*T221D.2*) at different timepoints after treatment (Supplementary Fig. S1).

**Fig. 6:**
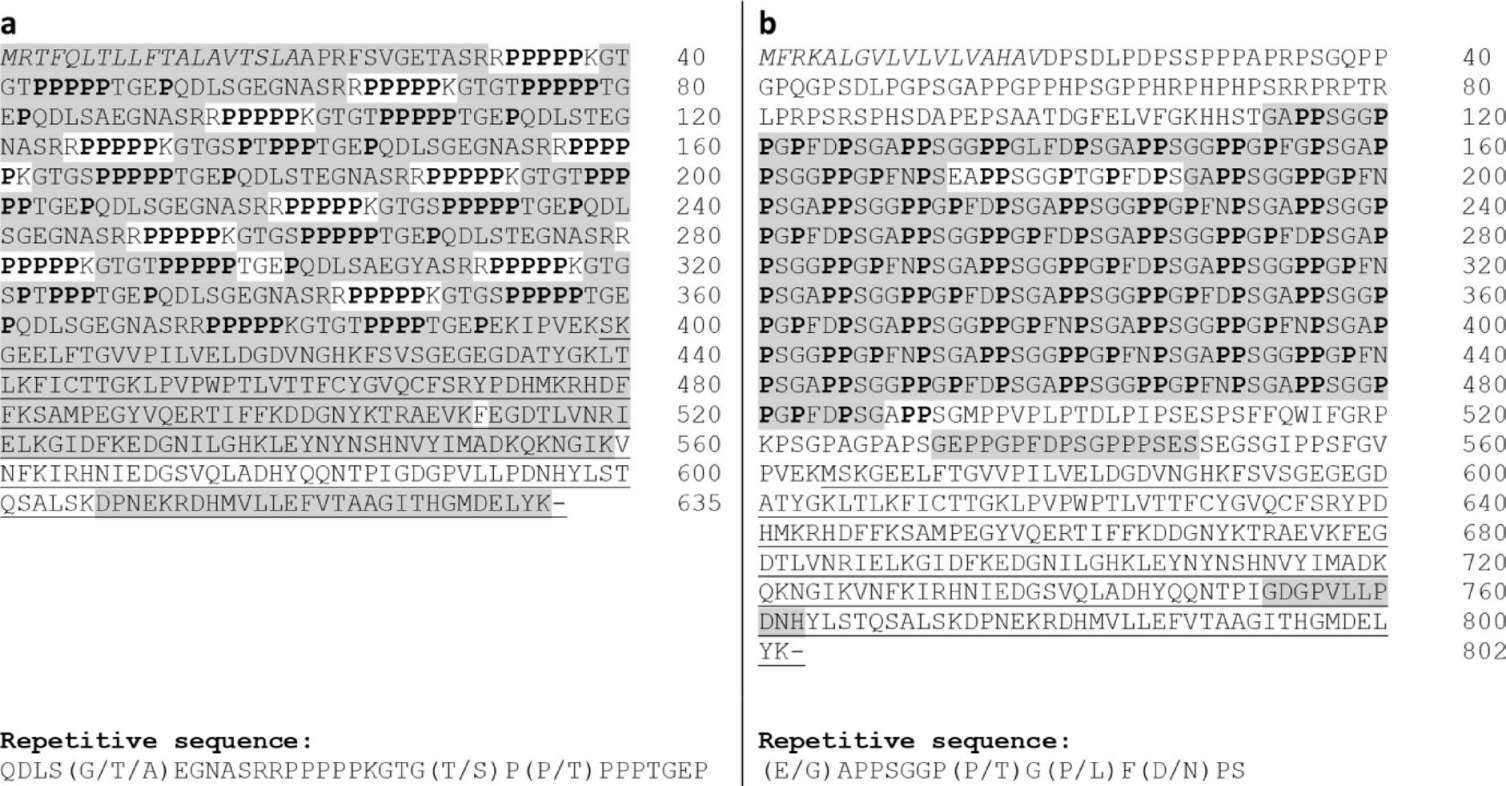
Protein sequences of (**a**) T22D1.2::GFP from WWU203 and (**b**) CLX-1::GFP from WWU202. Peptides detected by MS analysis are highlighted in grey. Sequences of signal peptides predicted by SignalP6.0^55^ are given in italics. Bold: Proline units within the repetitive part of the sequences. Underlined: Sequence of GFP.

In the next step, the corresponding proteins were isolated and their sequence analyzed, in order to verify that *T22D1.2* and *clx-1* were translated into proline-rich proteins. The respective GFP fusion proteins were obtained via GFP-Trap and their sequences were analyzed by mass spectrometry. Characteristic repetitive proline-rich fragments were detected in both of the isolated proteins (Fig. 6), indicating that the respective genes were indeed translated. Already during the course of plasmid construction for cloning, sequencing of the gene region of *T22D1.2* revealed a slight deviation from the anticipated gene sequence obtained from WormBase^54^. The hypothetical gene is located on chromosome IV (IV:6,921,878.6,922,109), but the respective sequence amplified from genomic DNA included two additional repeats within the highly repetitive region. Further, detection of an additional guanine base at 302 bp resulted in another repeat instead of a predicted intron (Supplementary Table S2).

**Fig. 7:**
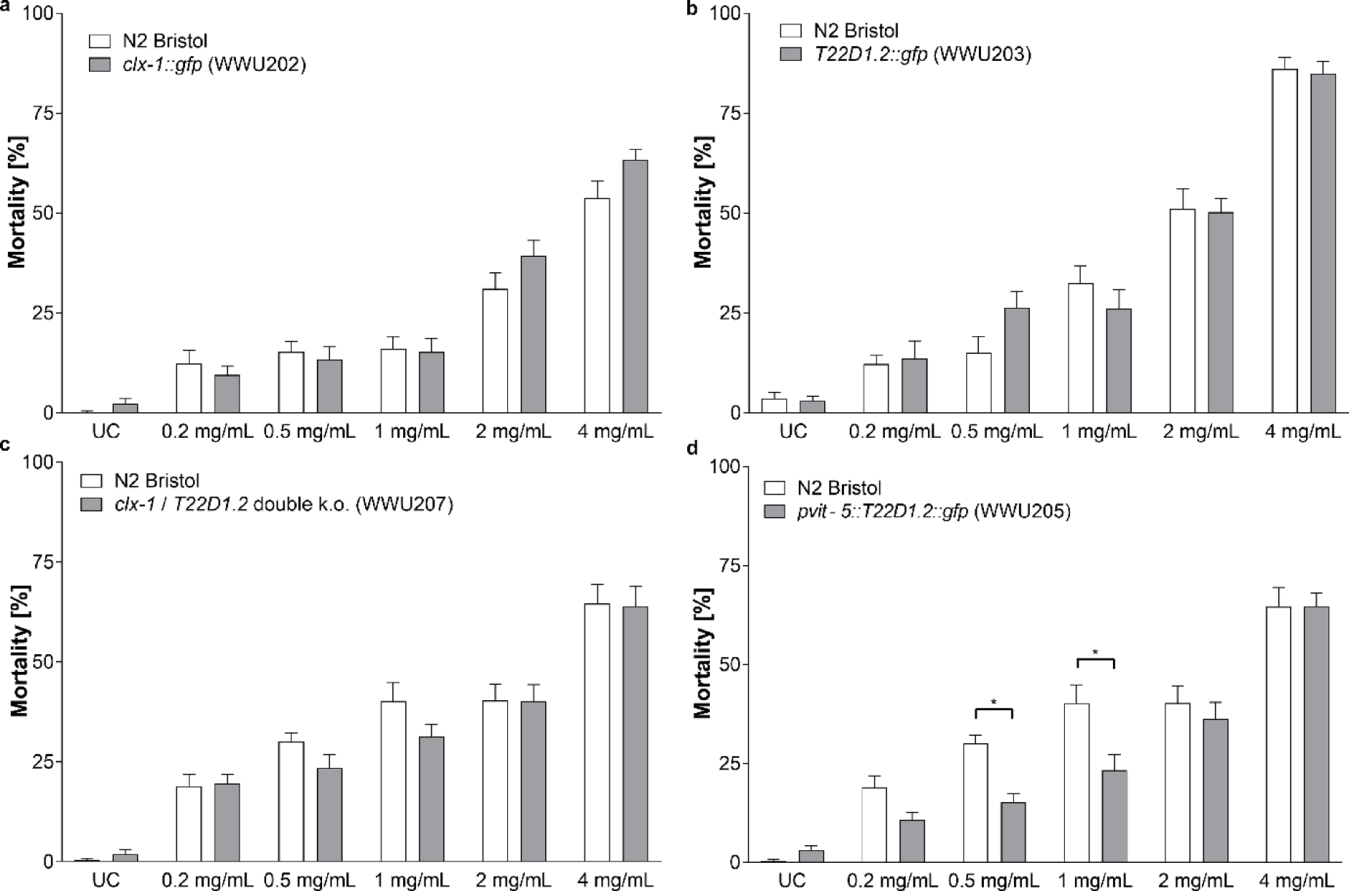
Mortality rates of different *C. elegans* strains after treatment with CM for 48 h at different concentrations compared to the wild-type N2 Bristol strain. No differences were observed for (**a**) worms overexpressing *clx-1* or (**b**) worms overexpressing *T22D1.2* as GFP fusion proteins, respectively. (**c**) Knockout of both genes, *clx-1* as well as *T22D1.2* did not lead to an increase in mortality. (**d**) Expression of *T22D1.2* under a stronger promoter (*pvit-5*) significantly enhanced resistance towards treatment with CM at lower concentrations. Bars represent means ± SEM. *: *p* < 0.05, determined by two-way ANOVA with Bonferroni’s test for multiple comparisons.

Due to their similarity to human basic salivary proline-rich proteins and their strong induction by tannins, we speculated that CLX-1 and T22D1.2 could represent a mechanism of defense. Therefore, the susceptibility towards CM was assayed in *C. elegans* overexpressing CLX-1 or T22D1.2 and in mutants that were deficient for the two proteins. Surprisingly, transgenic worms overexpressing either *clx-1* (Fig. 7a) or *T22D1.2* (Fig. 7b) did not show improved resistance towards treatment with CM. On the other hand, the susceptibility of knockout mutants was not increased, either, compared to the wild-type (Supplementary Fig. S2), not even in the *clx-1*/*T22D1.2* double knockout mutant (Fig. 7c). However, when T22D1.2 was constitutively expressed under the strong *vit-5* promoter, resistance in strain WWU205 significantly increased at lower concentrations *versus* the wild-type strain (Fig. 7d).

**Fig. 8:**
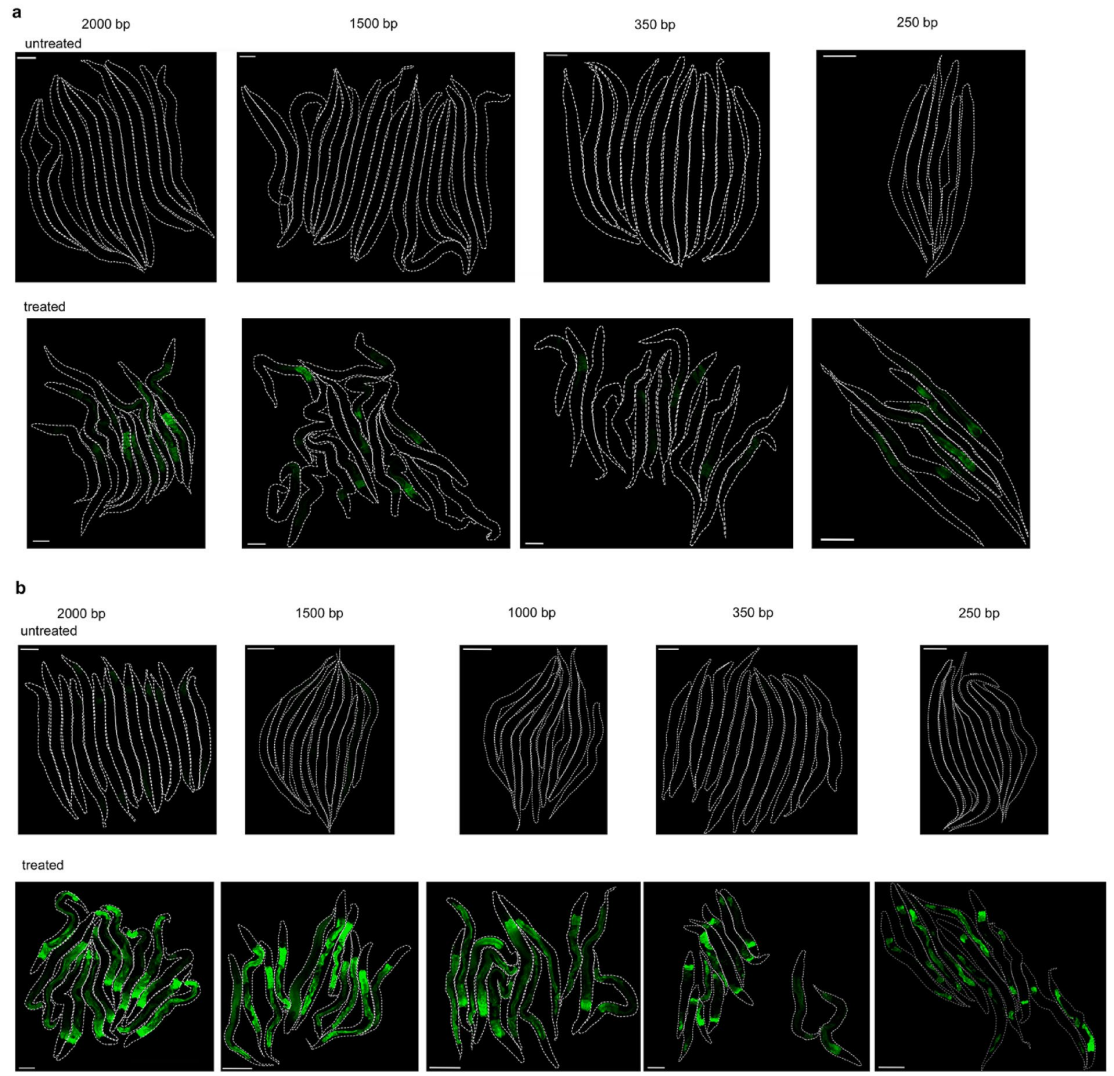
Expression of GFP in *C. elegans* controlled by promoter fragments of different length. (**a**) Fragments of 2000 bp, 1500 bp, 350 bp and 250 bp upstream of *T22D1.2* did not lead to fluorescence in untreated animals (top), but induced GFP expression after treatment with 0.2 mg/mL CM for 18 h (bottom). (**b**) Fragments of 2000 bp, 1500 bp, 1000 bp, 350 bp and 250 bp upstream of *clx-1* did not lead to detectable expression of GFP under the applied acquisition settings in untreated animals (top), but GFP expression was induced by treatment with 0.2 mg/mL CM (bottom). Scale bar 100 μm. Experiments were performed in triplicate with at least ten animals per replicate.

To investigate the regulatory mechanisms of *clx-1* and *T22D1.2* and identify potential regulatory elements in their promoter regions, promoter fragments of varying lengths were fused to GFP. While no fluorescence was detected at all in the untreated groups, treatment with CM induced the expression of GFP (Fig. 8a, b). With altered microscope settings using a higher detector gain unsuitable for the treated group, also untreated worms with GFP expression under the *clx-1* promoter were brightly fluorescent (Supplementary Fig. S3).

## Discussion

Two genes, *clx-1* and *T22D1.2* that had previously been shown to be strongly induced in *C. elegans* exposed to condensed tannins^23^ were further characterized in this study. Both were found to be translated into proteins consisting of a signal peptide, a highly repetitive proline-rich region and a short non-repetitive sequence before the proline-rich repeats and the C-terminus of the protein. Such a sequence is strongly reminiscent of proline-rich proteins in different mammalian species^56,57^, although the amino acid composition of the repeats varied considerably in the worm. Further, both proteins are predicted to be orthologs of basic salivary proline-rich proteins in humans (T22D1.2: PRB4 and CLX-1: PRB1)^58^. Similar to the expression pattern observed in our study, PRPs are not constitutively expressed in rats or mice, but induced after administration of tannin-rich feeds^8,59^. GFP fusions of both proteins were detected in the worms’ intestine, suggesting they could confer a mechanism of defense against tannins ingested during food uptake from *C. elegans*’ natural surroundings. The observation that strain WWU205 (*vit-5p::T22D1.2::gfp*) was more resistant to the treatment with tannins supports this hypothesis. On the other hand, a knockout of both genes was expected to increase susceptibility of strain WWU207 compared to the wild type, but no difference was observed. Even if we assume that these PRPs possess a protective role, they may not represent the only mechanism of defense against tannins. Moreover, the parameter assessed was the mortality at concentrations that are typical for tannin-containing fruits^60,61^. However, tannins certainly exert detrimental effects in nematodes also at sublethal concentrations^62^. An induction of PRPs may therefore still confer protection which was not captured by the mortality assay. Despite their similarities in response to various stressors, there are certain differences between *T22D1.2* and *clx-1*. Regarding the protein sequence, CLX-1 also shares structural similarities with collagens, especially the glycine-proline-proline (GPP) motif^63,64^. However, in contrast to CLX-1, the GPP sequence is frequently found within non-repetitive regions of collagens^64^, and the tandem repeats occurring in CLX-1 are not characteristic for collagens, either. Still, they do not resemble those of salivary PRPs as strongly as the tandem repeats found in T22D1.2 with blocks of up to five proline residues^57^.

Concerning the regulation of the two genes, we did not identify a transcription factor or a motif within the promoter region that induces their transcription. In the transcriptional *clx-1* reporters with varying promoter fragments, GFP expression was generally detectable, whereas for T22D1.2, it was only induced upon tannin stress. In humans, macaques and several rodent species, salivary-specific cAMP-responsive elements (CRE) have been associated with the regulation of PRPs^56,65,66^. Although *C. elegans* is known to possess CREs^67^, we did not identify a respective characteristic motif in the promoter region. Previously, *clx-1* was found to be activated by the removal of germ line stem cells, but not by *skn-1*^68^. On the other hand, down-regulation of *clx-1* was observed during knockdown of *skn-1*^69^. Moreover, together with several other genes coding for cuticle collagens, *clx-1* was among the most strongly down-regulated genes following treatment of *C. elegans* with copper sulfate^70^, which is in contrast to our findings for T22D1.2. An up-regulation was reported in a *dpy-7* mutant showing disruptions of the furrows and, different to the current study, at high levels of sodium chloride, but only after 24 h of exposure^69^. Even if CLX-1 is not a collagen and was detected in the intestine, its co-expression with cuticle collagens and the impact of cuticle disruptions on its expression suggest that CLX-1 might not represent a direct mechanism of defense against tannins. Rather, it could be related to the destructive effects of tannins that bind to the nematode cuticle as their primary target^71,72^, although no alterations in the expression of cuticle collagens were observed in *C. elegans* after short-term treatment with condensed tannins^23^.

Regarding the microscopic images obtained in this study, it should be noted that fluorescence intensities generally varied extremely across the different samples, in line with the transcription of *clx-1* and T22D1.2 observed in the qPCR experiments. While in some samples, GFP expression was not observed at all, treatment with tannins led to a strong increase in fluorescence. Following general recommendations to avoid saturation^73–75^, it was therefore impossible to acquire all images under the same conditions. On the other hand, differences in the treated versus the control group were not always depicted accurately this way. We tried to account for this problem by including the microscopic data for both groups.

The current results further suggest that the expression of the two proline-rich proteins may be linked to the astringent properties of the test substances. In humans, the perception of astringency is a tactile sensation that seems to be mainly mediated by an interaction of tannins or other astringent molecules with salivary proteins such as PRPs, and a subsequent loss of lubrication of the oral mucosa^50,76^. If the sensation of astringency leads to an up-regulation of T22D1.2, we would also expect a stronger response to the exposure to metal ions. However, it could be that the tolerated concentrations of the respective salts, especially of aluminum sulfate, were too low to induce the expected effects. Additionally, susceptibility to and perception of astringency may vary between distantly related organisms, such as humans and nematodes. Elucidating the trigger for the induction of *clx-1* and *T22D1.2* in *C. elegans* as well as identification of the connecting elements between the collagenous, tannin binding cuticle and their up-regulation will be topics of further research.

In summary, our findings provide evidence that *T22D1.2* is translated into a PRP-like protein that seems to act in a similar way like salivary PRPs in mammals. Besides a set of yet functionally uncharacterized proline-rich effectors in the plant parasitic nematode *Pratylenchus penetrans*^21^, this is the first report of PRPs that putatively act as a mechanism of defense against tannins in invertebrates.

## Electronic supplementary material

Below is the link to the electronic supplementary material.

Supplementary Material 1

Supplementary Material 2

## Data Availability

All data generated or analysed during this study are included in this published article (and its Supplementary Information files).
