# Supplementary material for: Induction of proline-rich proteins in response to tannin treatment in Caenorhabditis elegans
Source: Sci Rep. 2025 Aug 11;15:29399. doi: 10.1038/s41598-025-11651-1 (PMC12340121; doi:10.1038/s41598-025-11651-1)
Supplement: Supplementary file 2 — Supplementary Material 2 [file 41598_2025_11651_MOESM2_ESM.docx]

*Supplementary Data*

Induction of proline-rich proteins in response to tannin treatment in *Caenorhabditis elegans*

Luise Greiffer^1^, Luka Ressmann^2^, Charlotte Sophia Kaiser^2^, Eva Liebau^2†^, Verena Spiegler^1†*^

^1^Institute for Pharmaceutical Biology and Phytochemistry, University of Münster, Münster, Germany

^2^Institute of Integrative Cell Biology and Physiology, University of Münster, Münster, Germany

^†^Contributed equally to project supervision

*Corresponding author

E-mail address: verena.spiegler@uni-muenster.de

Table S1: Sequences of primers used in the study.

| Construct / Function | Name | Sequence (5’→ 3’) | T_m_ (° C) |
| --- | --- | --- | --- |
| *clx-1p::clx-1::gfp* | IF_clx-1_pPD_KpnI_rev | CATTTTTTCTACCGGTACCCCAAATGACGGAGGAATTC | 70.5 |
| All clx-1 promoter constructs | IF_clx-1p_KpnI_rev | CATTTTTTCTACCGGTACCggTCTGAAATTCATATCAAATTG | 71.1 |
| *clx-1p::clx-1::gfp* | IF clx-1 pPD BamHI_fwd | CGACTCTAGAGGATCCAGCATATCACTTTCAGATAGG | 70.6 |
| *clx-1p::gfp* (2000 bp) | IF clx-1_pPD BamHI_for | CGACTCTAGAGGATCCAGCATATCACTTTCAGATAGG | 71.7 |
| *clx-1p::gfp* (1500 bp) | IF clx-1p 1522 bp BamHI_fwd | CGACTCTAGAGGATCC gatacattggtattgtataatctg | 70 |
| *clx-1p::gfp* (1000 bp) | IF clx-1p 1010 bp BamHI_fwd | CGACTCTAGAGGATCCgttgtaaatacacatttctg | 72 |
| *clx-1p::gfp* (350 bp) | IF clx-1p 353 bp BamHI_fwd | GTCGACTCTAGAGGATCCggtcaatgtaaataaagg | 71.1 |
| *clx-1p::gfp* (250 bp) | IF clx-1p 254 bp BamHI_fwd | GGTCGACTCTAGAGGATCCgttatcattagaaaacagtctg | 73 |
|  |  |  |  |
| *T22D1.2p::T22D1.2::gfp* | T22p_XbaI_GC_fwd | GCGCTCTAGAGCCAGAATGTTCAAGTTCG | 68.1 |
| *T22D1.2p::T22D1.2::gfp* | T22_AgeI_GC_rev | GCGCACCGGTATTTTCTCTGGCTCTC | 68 |
| Reverse primer for all T22D1.2 promoter constructs | 95T22D1.2PrKpnAS | tcattttttctaccggtacctgtgaattcaatgagtgatac | 71 |
| *T22p::gfp* 1500 bp | T22p 1505 bp BamHI fwd | GTCGACTCTAGAGGATCCtgcatatcctgaattc | 71 |
| *T22p::gfp* 1000 bp | IF T22p 1010 bp BamHI fwd | GTCGACTCTAGAGGATCCtctcattgcaattcac | 71.4 |
| *T22p::gfp* 350 bp | IF T22p 350 bp BamHI fwd | GTCGACTCTAGAGGATCCaatctgtagatgacactg | 71.7 |
| *T22p::gfp* 250 bp | IF T22p 257 bp BamHI fwd | GGTCGACTCTAGAGGATCCaacctagactattgacataatg | 71.3 |
| *vit-5p::T22D1.2::gfp* | pvit-5_T22_BamHI_fwd | GCGCGGATCCATGAGAACTTTCCAATTAAC | 66.8 |
| *vit-5p::T22D1.2::gfp* | pvit-5_T22_BamHI_rev | CGCGGATCCTATTTTCTCTGGCTCTCC | 68 |
| Removal of gfp start codon in *T22D1.2p::T22D1.2::gfp* by mutagenesis | Mut_pPD_GFP-ATG_fwd | TACCGGTAGAAAAAAGTAAAGGAGAAGAAC | 65.5 |
|  | Mut_pPD_GFP-ATG_rev | GTTCTTCTCCTTTACTTTTTTCTACCGGTA | 65.5 |
| Removal of gfp start codon in *vit-5p::T22D1.2::gfp* by mutagenesis | Mut_pvit:T22:GFP-ATG_fwd | GCCAGAGAAAATAGAACAGAGTAAAGGAGAAGAACTTTTCACTGG | 71.3 |
|  | Mut_pvit:T22:GFP-ATG_rev | CTCCTTTACTCTGTTCTATTTTCTCTGGCTCTCCAGTTGGTTG | 72.3 |
| Insertion of T22D1.2 sgRNA into pDD162 by 2-step mutagenesis | M1 T22ko_fwd | ttgcgagatgtcttGCAGAGAAGTTgttttagagctagaa | 69.5 |
|  | M1 T22ko_rev | ttctagctctaaaacAACTTCTCTGCaagacatctcgcaa | 69.5 |
|  | M2 T22ko_fwd | tcttGCAGAGAAGTTACCGCCAAAGgttttagagctagaa | 70.5 |
|  | M2 T22ko_rev | ttctagctctaaaacCTTTGGCGGTAACTTCTCTGCaaga | 70.5 |
| Cloning of T22D1.2 sgRNA in pRB1017 | T22D1.2 sgRNA_3_fwd | TCTTGTTTGGCGGTAACTTCTCTGG | 63 |
|  | T22D1.2 sgRNA_3_rev | AAACCCAGAGAAGTTACCGCCAAAC | 63 |
| Repair template | T22D1.2_KO HDR-Template | CTTTCCAATTAACACTACTCTTCACCGCTTTGGCGGTAACTTCTCTGTGACGTAGGTAGGTAGGATCCGCTCCGCGTTTCTCAGTTGGTGAGACTGCTTCTCGTCGTCCACCACC |  |
| Primers used for RT-PCR | | | |
| Amplification of *pmp‑3* | pmp-3 fwd | GTTCCCGTGTTCATCACTCAT | 57.9 |
|  | pmp-3 rev | ACACCGTCGAGAAGCTGTAGA | 59.8 |
| Amplification of *Y45F10D.4* | Y45F10D.4 fwd | GTCGCTTCAAATCAGTTCAGC | 57.9 |
|  | Y45F10D.4 rev | GTTCTTGTCAAGTGATCCGACA | 58.4 |
| Amplification of *T22D1.2* | T22D1.2 fwd | CCAATTAACACTACTCTTCACCG | 58.9 |
|  | T22D1.2 rev | GAAGCATTACCCTTCTTCTGCAGA | 61 |
| Amplification of *clx-1* | clx-1 fwd | ACATCCACATCCGTCGAGAA | 57.3 |
|  | clx-1 rev | TCAAACCCATCTGTTGCAGC | 57.3 |


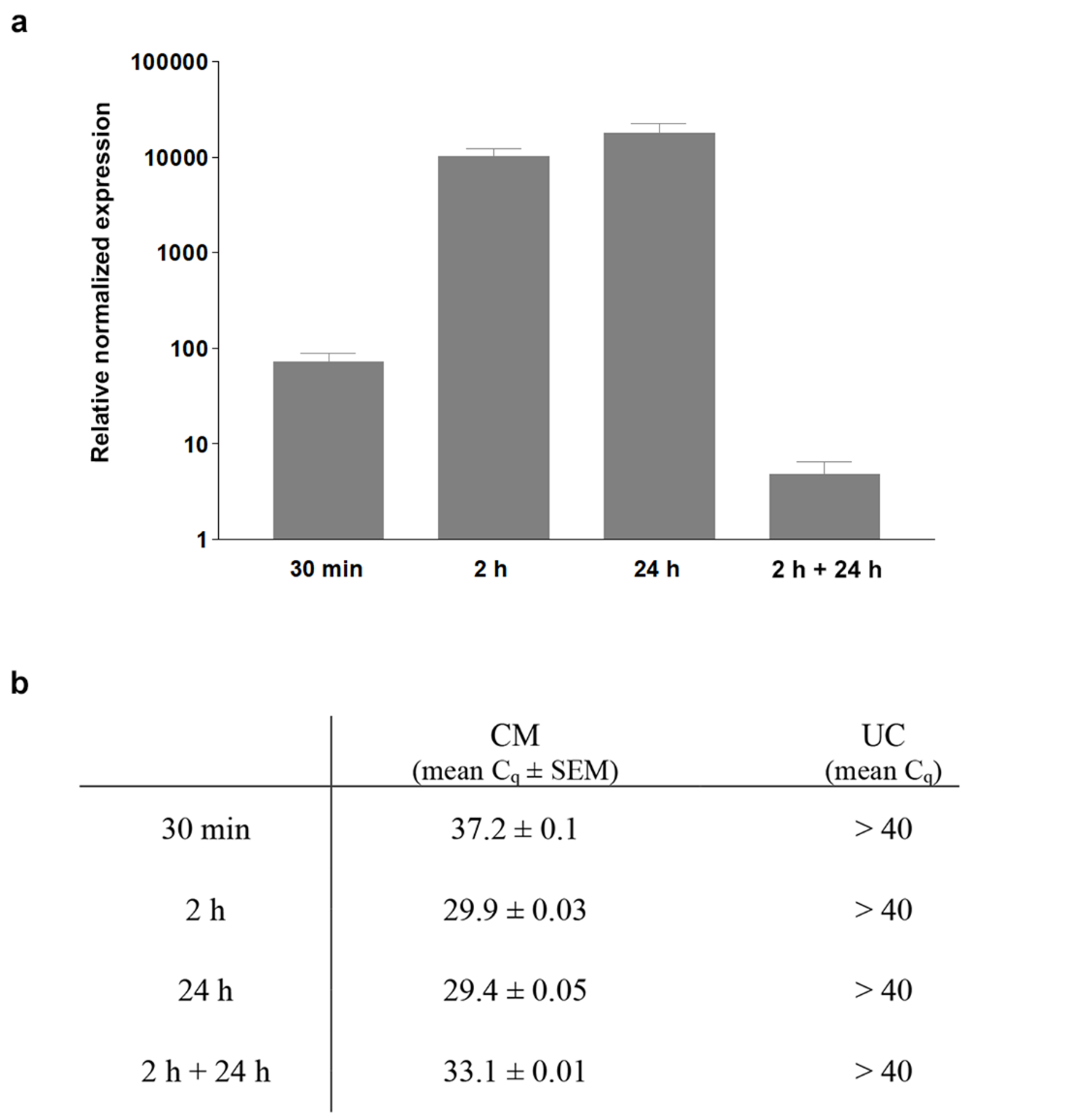


Figure S1: Gene expression analysis by RT-qPCR in wild-type *C. elegans* L4 larvae after treatment with 0.2 mg/mL CM in M9 buffer for 30 min, 2 h, 24 h, or for 2 h followed by 24 h of regeneration on NGM agar (2 h + 24 h). UC: untreated control (0.1 % DMSO in M9 buffer). (**a**) Mean relative normalized expression of *clx-1* determined by comparative *C*_T_ method. (**b**) Mean C_q_ values as an estimate for *T22D1.2* expression in treated worms versus the control. The low amount of transcript in the control group did not allow determination of the relative normalized expression. However, the data clearly indicate a strong induction by CM treatment.

Table S2: Gene sequence of *T22D1.2* obtained from sequencing the *T22D1.2.::gfp* reporter construct. Underlined: Repetitive sequence GCT TCT CGT CGT CCA CCA CCA/G CCA CCG AAG GGA ACT GGA AC/GC/T CCA A/CCA CCA CCA CCA ACT GGA GAA CCA CAG GAT CTT TCT A/GC/GA GAA GGT A/TAT. Bold: Start of each repeat. Highlighted in grey: Additional nucleotides compared to the sequence retrieved from WormBase [53].

| Gene sequence of *T22D1.2* (wormbase release WS255) [53] | Gene sequence of *T22D1.2* obtained in this study |  |
| --- | --- | --- |
| ATGAGAACTTTCCAATTAACACTACTCTTCACCGCTTTGGCGGTAACTTCTCTGGCGGCTCCGCGTTTCTCAGTTGGTGAGACT**G**CTTCTCGTCGTCCACCACCACCACCGAAGGGAACTGGAACTCCACCACCACCACCAACTGGAGAACCACAGGATCTTTCTGGAGAAGGTAAT**G**CTTCTCGTCGTCCACCACCACCACCGAAGGGAACTGGAACCCCACCACCACCACCAACTGGAGAACCACAGGATCTTTCTGCAGAAGgtaatgcttctcgtcgtccaccaccgccaccgaaggaactggaaccccaccaccaccaccaactggagaaccacaggatctttctacagAAGGTAAT**G**CTTCTCGTCGTCCACCACCACCACCGAAGGGAACTGGAA  CCCCACCACCACCACCAACTGGAGAACCACAGGATCTTTCTGGAGAAGGTAAT**G**CTTCTCGTCGTCCACCACCACCACCGAAGGGAACTGGAAGCCCACCACCACCACCAACTGGAGAACCACAGGATCTTTCTGGAGAAGGTAAT**G**CTTCTCGTCGTCCACCACCACCACCGAAGGGAACTGGAAGCCCACCACCACCACCAACTGGAGAACCACAGGATCTTTCTACAGAAGGTAAT**G**CTTCTCGTCGTCCACCACCGCCACCGAAGGGAACTGGAACCCCACCACCACCACCAACTGGAGAACCACAGGATCTTTCTGCAGAAGGTTAT**G**CTTCTCGTCGTCCACCACCACCACCGAAGGGAACTGGAAGCCCAACACCACCACCAACTGGAGAACCACAGGATCTTTCTGGAGAAGGTAAT**G**CTTCTCGTCGTCCACCACCACCACCGAAGGGAACTGGAAGCCCACCACCACCACCAACTGGAGAACCACAGGATCTTTCTGGAGAAGGTAAT**G**CTTCTCGTCGTCCACCACCACCACCGAAGGGAACTGGAACCCCACCACCACCAACTGGAGAGCCAGAGAAAATATAG | ATGAGAACTTTCCAATTAACACTACTCTTCACCGCTTTGGCGGTAACTTCTCTGGCGGCTCCGCGTTTCTCAGTTGGTGAGACT**G**CTTCTCGTCGTCCACCACCACCACCGAAGGGAACTGGAACTCCACCACCACCACCAACTGGAGAACCACAGGATCTTTCTGGAGAAGGTAAT**G**CTTCTCGTCGTCCACCACCACCACCGAAGGGAACTGGAACCCCACCACCACCACCAACTGGAGAACCACAGGATCTTTCTGCAGAAGGTAAT**G**CTTCTCGTCGTCCACCACCGCCACCGAAGGGAACTGGAACCCCACCACCACCACCAACTGGAGAACCACAGGATCTTTCTACAGAAGGTAAT**G**CTTCTCGTCGTCCACCACCACCACCGAAGGGAACTGGAA  GCCCAACACCACCACCAACTGGAGAACCACAGGATCTTTCTGGAGAAGGTAAT**G**CTTCTCGTCGTCCACCACCACCACCGAAGGGAACTGGAAGCCCACCACCACCACCAACTGGAGAACCACAGGATCTTTCTACAGAAGGTAAT**G**CTTCTCGTCGTCCACCACCGCCACCGAAGGGAACTGGAA  CCCCACCACCACCACCAACTGGAGAACCACAGGATCTTTCTGGAGAAGGTAAT**G**CTTCTCGTCGTCCACCACCACCACCGAAGGGAACTGGAAGCCCACCACCACCACCAACTGGAGAACCACAGGATCTTTCTGGAGAAGGTAAT**G**CTTCTCGTCGTCCACCACCACCACCGAAGGGAACTGGAAGCCCACCACCACCACCAACTGGAGAACCACAGGATCTTTCTACAGAAGGTAAT**G**CTTCTCGTCGTCCACCACCGCCACCGAAGGGAACTGGAACCCCACCACCACCACCAACTGGAGAACCACAGGATCTTTCTGCAGAAGGTTAT**G**CTTCTCGTCGTCCACCACCACCACCGAAGGGAACTGGAAGCCCAACACCACCACCAACTGGAGAACCACAGGATCTTTCTGGAGAAGGTAAT**G**CTTCTCGTCGTCCACCACCACCACCGAAGGGAACTGGAAGCCCACCACCACCACCAACTGGAGAACCACAGGATCTTTCTGGAGAAGGTAAT**G**CTTCTCGTCGTCCACCACCACCACCGAAGGGAACTGGAACCCCACCACCACCAACTGGAGAGCCAGAGAAAATATAG | 37  74  111  148  185  222  259  296  333  370  403  440  477  514  551  588  589  626  663  700  737  774  811  848  885  922  959  996  1033  1070  1107  1144  1181  1185 |


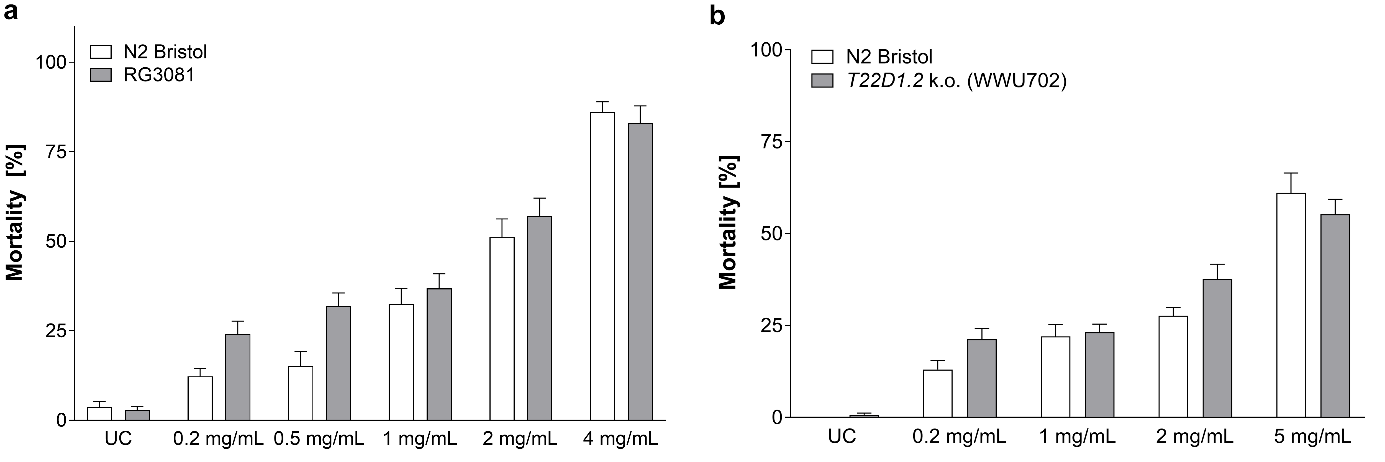


Figure S2: Mortality rates of different *C. elegans* strains after treatment with CM for 48 h at different concentrations compared to the wild-type N2 Bristol strain. No differences were observed for (**a**) strain RG3081 carrying a deletion of *clx-1* or (**b**) in *T22D1.2* knockout strain WWU702. Bars represent means ± SEM.


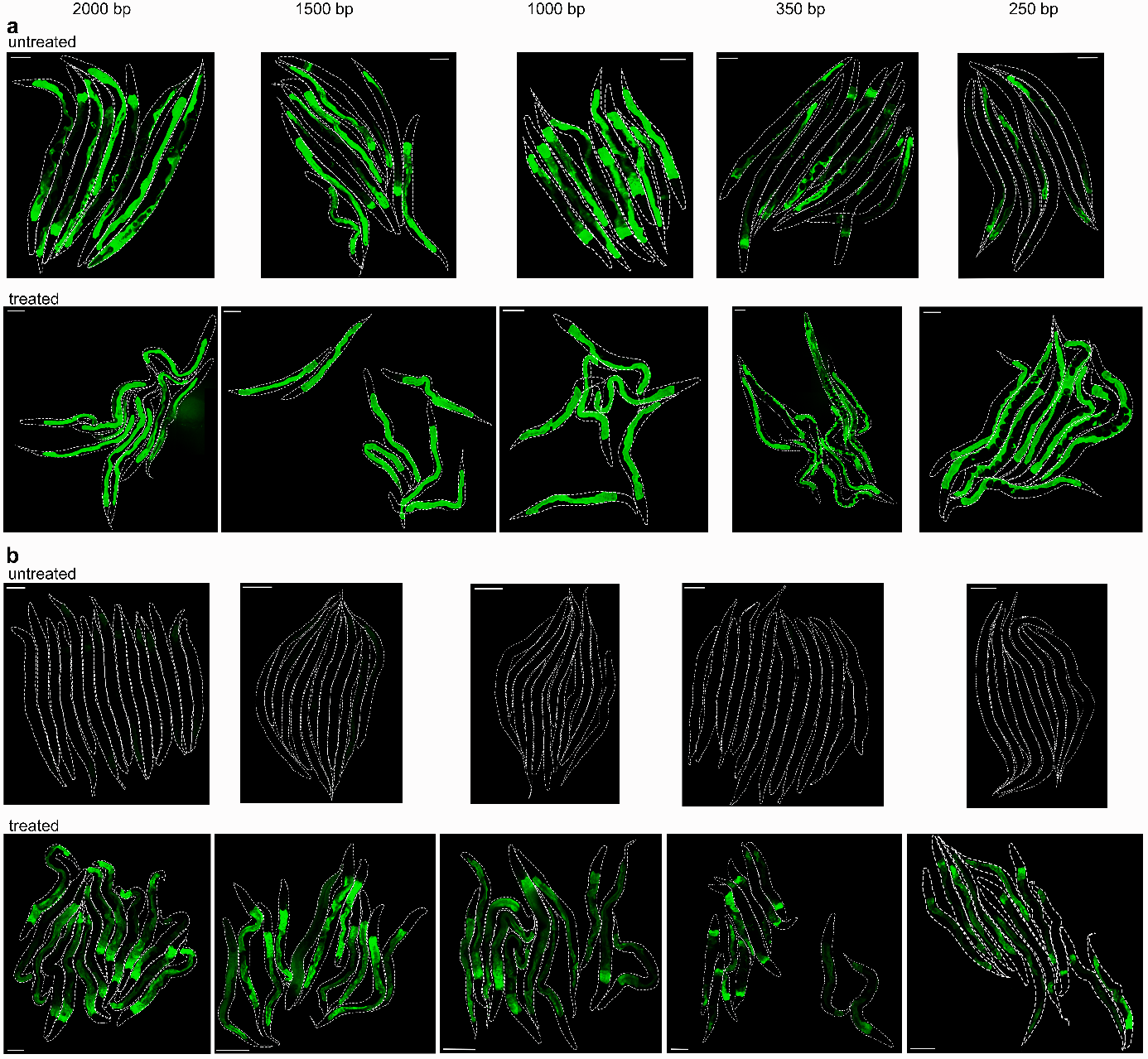


Figure S3: Comparison of the different image acquisition settings to monitor the expression of GFP in *C. elegans* controlled by promoter fragments of 2000 bp, 1500 bp, 1000 bp, 350 bp and 250 bp upstream of *clx-1*. (**a**) Images acquired with the detector gain set to 500 resulted in fluorescent untreated and treated worms. (**b**) Identical to Figure 8b in the main text: In order to avoid detector saturation, the gain was decreased to 350 and out-of-focus light was reduced. However, GFP expression in the untreated control group was not detectable under these conditions. Scale bar 50 µm.
